# A Case Report of Non-puerperal Uterine Inversion due to Submucosa Leiomyoma in a Young Virgin Woman

**DOI:** 10.1155/2022/5240830

**Published:** 2022-08-16

**Authors:** Faezeh Moshayedi, Hengameh Sadat Seidaei, Amir Mohammad Salehi

**Affiliations:** ^1^Clinical Research Development Unit of Fatemiyeh Hospital, Department of Gynecology, Hamadan University of Medical Sciences, Hamadan, Iran; ^2^School of Medicine, Hamadan University of Medical Sciences, Hamadan, Iran

## Abstract

The inversion of the uterus is a rare complication of the puerperium and much rarer during the non-puerperal period. The most common cause of non-puerperal inversion is a submucosa leiomyoma; however, identification can be difficult. Herein, we present a case of pedunculated uterine leiomyoma that causes uterus inversion in a 30-year-old virgin woman with a correct diagnosis; a hysterectomy was prevented for the patient.

## 1. Introduction

Uterine leiomyoma (UL) is the most common benign tumor of the female genital tract and has become a major health problem [1]. UL occurs in 20 to 40% of women of reproductive age and is the major cause of hysterectomy. [2] Based on their location in the uterus, UL are classified as subserosal, intramural, and submucosal. Some submucosal UL become peduncles in the uterine cavity and can dilate the cervix, protrude from the vagina, and become necrotic and infected.

UL can have many side effects such as dyspareunia [3], increase the risk of endometriosis, postpartum hemorrhage, placenta previa, and reduced quality of life [4–7]. Uterine inversion (UI) is one of the rare complications of ULs. UI is briefly depicted as the indentation and depression of the fundus extending downwards up to the various levels of the vaginal canal [8]. Herein, we present a case of pedunculated UL causes UI in a 30-year-old virgin woman.

## 2. Case Reports

Our case report describes a 30-year-old virgin woman presented at the emergency room of Fatemieh Hospital with vaginal bleeding and a protrusion of a mass from the vagina. The patient had a history of severe bleeding in the form of hypermenorrhea, which had intensified the day before the emergency room visit and also felt a feeling of pressure in the perineum and a mass coming out of the vagina. The patient was conscious during the physical examination.

The physical examination revealed a body temperature of 37 °C, a heart rate (HR) of 105 beats per minute, a respiratory rate (RR) of 18 beats per minute, and a blood pressure (BP) of 90/60 mmHg. Pelvic examination revealed the presence of a large mass measuring 6-7 cm in diameter with a necrotic appearance and protruding from the cervix and vagina. Necessary tests were requested for the patient (Table 1). Due to lower blood pressure and low hemoglobin levels, the patient was first resuscitated with two blood bags and then with suspicion of a peduncle UL transferred to the operating room (OR).

**Figure 1:**
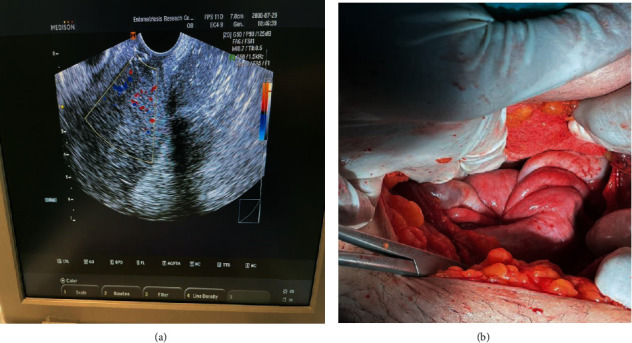
Ultrasound findings indicate the absence of a uterus in the pelvic cavity (a). Confirmation of the absence of uterus in the abdominal cavity after abdominal incision (b).

The vagina was examined in the OR after induction of anesthesia. Due to the abnormal thickness and excessive size of the UL pedicel, the UI was suspected following the protruding of the UL from the vagina, so the patient was asked for an ultrasound. Abdominal ultrasound of the uterus in the pelvic cavity was not observed and the diagnosis of UI was confirmed (Figures 1(a) and 1(b)).

**Figure 2:**
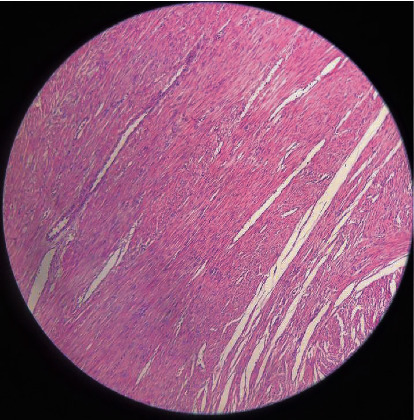
Pathology finding showed hemorrhagic leiomyoma with ulcerate and infected surface.

Therefore, myomectomy was performed carefully from the fundus level for the patient, and samples were sent for pathological examination (Figure 2). The endometrium below the UL was repaired with a 2-0 chromic suture, and then the abdomen was opened with a Pfannenstiel incision.

**Figure 3:**
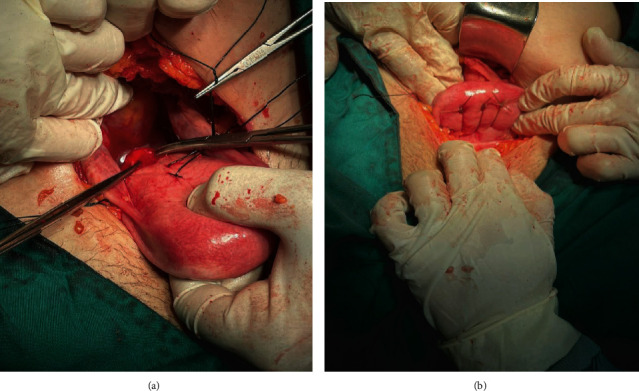
The uterus was returned to the pelvic cavity (a) and then uterine suspension to the round ligament (b).

The uterus was returned to the cervix and pelvis, and then the uterine suspension was performed to prevent UI to the round ligament (Figures 3(a) and 3(b)). The abdomen was washed and closed. 2 days later, the patient was discharged in good condition. Six months later, the patient's menses were regular, and the patient's dysmenorrhea was resolved. Also, the patient did not report any special problems; currently, the patient is married and about to become pregnant.

## 3. Discussion

UI are classified into two groups, including (a) puerperal and (b) non-puerperal inversions [9]. Non-puerperal UI is rare condition and the diagnosis can be difficult even on physical examination [10] In general, non-puerperal uterine inversion presents after 45 years and is mostly related to UL and seldom associated with malignancies [10]. In our patient, UI was also difficult to diagnose and was initially thought to be just a peduncle UL.

The etiology of UI is not well understood. A thin uterine wall, rapid tumor growth, tumor size, fundic localization of the tumor, tumor attachment to the uterine wall with a thin pedicle, dilatation of the cervix due to uterine cavity distension, and quick expulsion of the tumor are all possible factors [11]. Most non-puerperal UI are caused by benign submucosa UL, while other causes are rhabdomyosarcoma, leiomyosarcoma, and endometrial polyp [10]. Anemia caused by irregular vaginal bleeding, vaginal discharge, lower abdomen and/or pelvic pain, a protruding mass in the vagina, and, in some circumstances, obstruction of the urethra are the most common symptoms of non-puerperal UL [10].

Imaging techniques such as ultrasound and magnetic resonance imaging (MRI) help with the diagnosis. However, the diagnostic value of transvaginal ultrasound is limited, especially in cases where large mass protrude into the vagina, because of physical limitations; the probe cannot be inserted into a choked vaginal canal; another issue is that ultrasonic beams could not be transferred beyond the mass, resulting in low-quality sonographic images [12]. Therefore, MRI is the preferred diagnostic tool and can provide an accurate diagnosis before surgery [12] Due to the lack of MRI in our hospital, an ultrasound was performed for the patient.

Reposition procedures, according to the reproductive desire of the patient, or hysterectomy could be considered for surgical treatment [12]. In our patient, due to nulliparous and incomplete family plan, hysterectomy was not performed, and the uterus was returned to the pelvic cavity with surgical techniques. Unwanted hysterectomy was also prevented due to the correct diagnosis of UI due to peduncle UL.

## Figures and Tables

**Table 1:** Laboratory test.

Test	Result	Test	Normal range
WBC	12000	/uL	4000-10000
RBC	3.4	10^6^/uL	3.9-5.6
Hemoglobin	6	g/dL	11.5-16.5
Hematocrit	23.1	%	36-47
Platelets	288	10^3^/uL	150-450
MCV	67.9	IL	80-100
MCH	17.6	Pg	27-32
MCHC	26	g/dL	32-36
PT	12.4	S	11.5-13
INR	1.1	S	1
Creatinine	0.98	Mg/dL	0.5-1.5
Fibrinogen	380	Mg/dL	150-350

## Data Availability

Access to data is permitted with the author's permission.
